# When should a neonatologist consult a rheumatologist?

**DOI:** 10.1007/s00431-025-06086-9

**Published:** 2025-03-18

**Authors:** Ali Öksel, Nihal Şahin, Ayla Günlemez

**Affiliations:** 1 Medicalpark Hospital, Pediatrics, Kocaeli, Turkey; 2 Department of Pediatric Rheumatology, Kocaeli University, Kocaeli, Turkey; 3 Department of Neoanatology, Kocaeli University, Kocaeli, Turkey

**Keywords:** Autoinflammatory diseases, Maternal, Medication on pregnancy, Neonates, Rheumatic diseases

## Abstract

Pediatric rheumatologic diseases are complex conditions that can present with various clinical manifestations, including fever, rash, joint involvement, and diarrhea, impacting more than one organ system and affecting all pediatric age groups from 0 to 18 years. This review focuses on rheumatologic diseases in neonates, encompassing both primary neonatal-onset conditions and those influenced by maternal autoimmune diseases and treatments during pregnancy. Diagnosing rheumatologic diseases in neonates is challenging due to their nonspecific symptoms, which can overlap with other conditions. While primary neonatal-onset diseases such as cryopyrin-associated periodic syndromes (CAPS), deficiency of IL-1 receptor antagonist (DIRA), and neonatal-onset juvenile idiopathic arthritis (JIA) are rare, maternal autoimmune diseases and their treatments can also impact neonatal health. Conditions like systemic lupus erythematosus (SLE) and antiphospholipid syndrome (APS) may increase neonatal risks, leading to complications such as thrombosis or pregnancy loss. Identifying these conditions early and providing the proper care is crucial to reduce morbidity and mortality in this vulnerable group.

*Conclusion*: Persistent fever, rash, or unexplained joint involvement warrants early referral to a pediatric rheumatologist. A multidisciplinary approach involving obstetricians, rheumatologists, and neonatologists is essential for timely diagnosis and optimal neonatal outcomes.

**Table:** 

What is Known: *• Diagnosis of neonatal rheumatologic diseases is difficult because their symptoms are nonspecific and may overlap with other neonatal diseases.* *• Maternal autoantibodies transmitted through the placenta may lead to neonatal complications (e.g. congenital heart block, thrombosis).* What is New: *• Long-term follow-up of autoinflammatory diseases is essential, as the absence of neonatal-specific damage indices limits the ability to assess disease progression and treatment outcomes, underscoring the need for validated scoring systems tailored to neonates.* *• Novel biomarkers, such as elevated levels of cord C-reactive protein, NT-proBNP, MMP-2, uPA, uPAR, and plasminogen, have been identified, offering new insights into potential diagnostic tools for cardiac neonatal lupus.*

What is Known:

*• Diagnosis of neonatal rheumatologic diseases is difficult because their symptoms are nonspecific and may overlap with other neonatal diseases.*

*• Maternal autoantibodies transmitted through the placenta may lead to neonatal complications (e.g. congenital heart block, thrombosis).*

What is New:

*• Long-term follow-up of autoinflammatory diseases is essential, as the absence of neonatal-specific damage indices limits the ability to assess disease progression and treatment outcomes, underscoring the need for validated scoring systems tailored to neonates.*

*• Novel biomarkers, such as elevated levels of cord C-reactive protein, NT-proBNP, MMP-2, uPA, uPAR, and plasminogen, have been identified, offering new insights into potential diagnostic tools for cardiac neonatal lupus.*

## Introduction

Pediatric rheumatologic diseases are complex conditions characterized by diverse symptoms, including fever, rash, joint involvement, and diarrhea, affecting multiple organ systems and occurring from the neonatal period to adolescence. These diseases require a broad multidisciplinary approach, with treatment strategies varying according to the disease’s type, course, and extent. While they are more commonly observed during adolescence, they can also present in early childhood and even in the neonatal period [1].

Rheumatologic diseases primarily encompass groups of disorders driven by autoinflammatory and autoimmune mechanisms. The maturation of the neonatal immune system relies on a balance between Th1 and Th2 responses. Th1-type cytokines drive pro-inflammatory reactions to eliminate intracellular pathogens, whereas Th2-type cytokines promote anti-inflammatory responses to counteract Th1-mediated bactericidal activity. In rare cases, fever occurring within the first weeks of life may indicate an autoinflammatory condition. Neonatologists should consider autoinflammatory syndromes, a growing group of genetic disorders affecting the innate immune system, primarily mediated by myeloid cells [2]. The difficulty in interpreting symptoms in newborns, combined with the potential for coexisting conditions to obscure underlying rheumatologic diseases, makes neonatal rheumatologic disorders a challenging group of diseases. These conditions require careful attention, as they present diverse clinical manifestations and necessitate a multidisciplinary approach to management and treatment. Autoinflammatory diseases can be seen as primary rheumatologic diseases in newborns, as well as conditions related to maternal rheumatologic diseases affecting the newborn. During pregnancy, overall autoimmune tolerance generally increases. However, while some autoimmune diseases go into remission, others may flare up. Rheumatoid arthritis (RA) often tends to improve during pregnancy. The increase in estrogen and progesterone levels may contribute to suppressing pro-inflammatory cytokines, reducing symptoms. Systemic lupus erythematosus (SLE) is an autoimmune disease with a high risk of flaring during pregnancy [3]. Hormonal changes can trigger lupus symptoms. The presence of antiphospholipid antibodies (aPL) or antiphospholipid syndrome (APS) can increase the risk of thrombosis and lead to complications such as recurrent miscarriages. Changes in thyroid function during pregnancy may exacerbate autoimmune thyroid diseases, such as Graves’ disease. Diseases like Crohn’s disease and ulcerative colitis may sometimes flare up during pregnancy [4].

Maternal conditions such as SLE, primary Sjögren’s syndrome (pSS), RA, dermatomyositis/polymyositis (DM/PM), and systemic sclerosis (SSc) are the most common causes of neonatal/fetal involvement. It is likely that maternal immune dysregulation during pregnancy, preeclampsia, and existing autoimmune diseases contribute to placental insufficiency. The direct transfer of immunoglobulin-G-class autoantibodies from the mother has also been implicated. Furthermore, medications used by the mother to manage her condition can also lead to certain complications in the child [5].

Infants born to mothers with rheumatic diseases are at risk for conditions such as small for gestational age (SGA) and intrauterine growth restriction (IUGR). Consequently, vigilant postnatal monitoring of their growth and development is paramount. Studies have shown that catch-up growth in infants born to mothers with rheumatic diseases occurs around 2 years of age compared to normal infants. As these infants grow, the *z*-scores for body weight and height progressively increase [5, 6].

This review will explore autoinflammatory diseases in neonates, along with conditions associated with maternal autoimmune rheumatologic diseases and the use of anti-rheumatic medications during pregnancy (see Box 1). Recognizing these conditions—which can present with diverse symptoms—is crucial for early diagnosis and treatment, ultimately reducing morbidity and mortality.

**Box 1** Rheumatological diseases affecting newborns

**Table:** 

Rheumatological diseases affecting newborns	
Autoinflammatory Diseases occurring in the neonatal period	Maternal autoimmune rheumatologic diseases in neonates
Neonatal-Onset Multisystem Inflammatory Disease (NOMID) Deficiency of IL-1 Receptor Antagonist Deficiency of Interleukin-36 Receptor Antagonist Majeed Syndrome Interpheronopathies Granulomatous and Autoimmune-Like Disorders Blau Syndrome Nuclear Factor- kB-Related Disorders Neonatal Kawasaki Disease Neonatal Behçet’s Disease Neonatal-Onset Juvenile Idiopathic Arthritis	Maternal SLE and Neonatal Lupus Maternal APL Positivity/Neonatal Anti-Phospholipid Antibody Syndrome Maternal Primary Sjögren Syndrome Maternal Rheumatoid Arthritis Maternal Dermatomyositis/Polymyositis Maternal Systemic Sclerosis

## Autoinflammatory diseases occurring in the neonatal period

The newborn’s immune system develops from birth and is influenced by genetic, epigenetic, and environmental factors. A balance between pro-inflammatory Th1 and anti-inflammatory Th2 responses marks its maturation [7]. Early immune imprinting can have long-term health effects, with breastfeeding playing a crucial role in this process [8]. In rare cases, fever in the first weeks of life may indicate autoinflammatory syndromes, a group of genetic disorders involving the innate immune system characterized by recurrent inflammatory episodes and systemic inflammation. A limited number of autoinflammatory syndromes may manifest in the neonatal period, presenting as antibiotic-resistant sepsis or nonbacterial osteomyelitis [9].

Although systemic autoinflammatory diseases are rare, they can result in significant morbidity and mortality if not treated. Uncontrolled disease activity may result in organ damage, disability, and reduced quality of life, placing a significant burden on both patients and their families. While damage indices exist for older age groups, there is a lack of validated tools for newborns [10]. Early diagnosis and targeted treatment are crucial to prevent long-term complications, highlighting the need for neonatal-specific diagnostic and prognostic scoring systems. Future research should focus on developing tailored assessment tools to improve outcomes in this vulnerable population.

### Neonatal-onset multisystem ınflammatory disease (NOMID)

Neonatal-onset multisystem inflammatory disease (NOMID), also known as chronic infantile neurological cutaneous and articular syndrome (CINCA), is an interleukin-1 (IL-1)-related autoinflammatory disorder. Pathogenic variants in NLRP3 on chromosome 1q44 are responsible for these conditions, which are generally inherited in an autosomal dominant manner. Mutations in NLRP3 lead to inappropriate production of IL-1 beta and IL-18, characterized by an increased inflammatory response [11–13].

Neonatal-onset multisystem inflammatory disease, also referred to as CINCA, is the most severe form of CAPS. It is typically associated with symptoms such as urticarial migratory rash, fever, growth and developmental delays, and facial abnormalities like frontal bossing and nasal deformities present at birth. This condition can lead to severe complications, including meningitis, sensorineural hearing loss, cerebral atrophy, uveitis, lymphadenopathy, hepatosplenomegaly, and potentially secondary amyloidosis, which can cause premature death [14].

### DIRA (deficiency of IL-1 receptor antagonist)

This condition manifests as sterile multifocal osteomyelitis, periostitis, and neutrophilic pustulosis in newborns, typically without fever. If left untreated, it may progress to multiple organ dysfunction syndrome. Medications such as anakinra, rilonacept, and canakinumab can be administered. The efficacy of thalidomide and tocilizumab is also under investigation [15].

### DITRA (deficiency of ınterleukin-36 receptor antagonist)

Interleukin-36 receptor antagonist deficiency (DITRA) is a rare and potentially life-threatening autoinflammatory disorder characterized by widespread erythematous and pustular skin eruptions, often accompanied by periodic fever. This condition is inherited autosomal recessive and results from mutations in the IL-36RN gene located on chromosome 2q13. The absence of a functional IL-36 antagonist leads to excessive binding of IL-36 cytokines to their receptors, causing persistent activation of inflammatory responses via the NF-κB and MAPK signaling pathways. Treatment with biological agents, including anakinra, has shown efficacy in patients with severe cutaneous and systemic manifestations [16].

### Majeed syndrome

Majeed syndrome is a multisystemic autosomal recessive disorder characterized by chronic recurrent multifocal osteomyelitis, recurrent bone pain, periodic fever, growth retardation, dyserythropoietic anemia, and neutrophilic dermatosis. It is caused by mutations in the LPIN2 gene, which encodes the enzyme lipin-2, a phosphatidic acid phosphatase. The syndrome is very rare. Severe cases presenting with recurrent fever, significant multifocal osteomyelitis, growth retardation, and markedly elevated inflammatory markers have been reported. The disruption of phosphatidic acid phosphatase activity in LIPIN2 leads to abnormal activation of the NLRP3 inflammasome and excessive production of proinflammatory cytokines, including IL-1β [17, 18]. Interleukin-1 blockers can be used in treatment, with reports in the literature of successful outcomes using Canakinumab or Anakinra [19].

### Interferonopathies

Type 1 interferons, including IFN-α and IFN-β, are essential components of the innate immune system, produced in response to viral infections and playing a vital role in controlling these infections. Interferonopathies are genetic conditions that cause excessive, spontaneous production of type 1 interferons, leading to autoinflammatory responses. These disorders typically arise from defects in pathways involved in detecting or processing intracellular nucleic acids—molecules often associated with viral infections—resulting in continuous type 1 interferon production. Both monogenic and polygenic forms of interferonopathies have been identified, with some presenting as early as the neonatal period. Due to their symptoms, these disorders can sometimes be confused with autoimmune diseases like systemic lupus erythematosus, which also displays a disrupted interferon signature. Interferons activate receptors that rely on Janus kinase (JAK) proteins for signaling, and recent advancements with JAK inhibitors have shown promise in treating these conditions [20, 21].

STING-associated vasculopathy with onset in infancy (SAVI) is a rare autoinflammatory disease caused by gain-of-function pathogenic variants in TMEM173, which encodes STING (stimulator of interferon genes). STING, an endoplasmic reticulum protein, plays a crucial role in activating the type I interferon (IFN) response. Upon IFN receptor binding, Janus kinases (JAKs) are activated, leading to the upregulation of interferon-stimulated genes. Clinically, SAVI manifests with early-onset systemic inflammation, characteristic skin involvement (painful cutaneous ulcers, telangiectasias, and livedo reticularis), and progressive interstitial lung disease. Additional features include recurrent fever, myositis or myalgia, and arthritis or arthralgia. Among these, the severity of lung involvement is the primary determinant of disease prognosis.

### Granulomatous and autoimmune-like disorders

#### Blau syndrome

Blau syndrome (BS) is a dominantly inherited granulomatous disorder characterized by chronic dermatitis, symmetric arthritis, and recurrent uveitis, often accompanied by fever. It is caused by gain-of-function mutations in the NOD2 gene, leading to NF-κB activation and excessive IL-1 and TNF secretion. Skin involvement is the earliest and most common feature, sometimes presenting as a granulomatous dermatitis in the neonatal period. Joint symptoms typically develop later, with hypertrophic tenosynovitis, while severe panuveitis is the most serious complication, potentially causing vision loss. Treatment aims to prevent ocular and articular damage, with NSAIDs for mild cases and corticosteroids for more severe forms. Immunosuppressants and biological agents such as infliximab or anakinra may be considered in refractory cases, though evidence on their efficacy remains limited [9, 22].

#### Nuclear factor-kB-related disorders

Various cytokine receptors and immune pathways can activate nuclear factor-kB (NF-kB), a key regulator of inflammatory responses. Mutations in two enzymes, otulin and A20, are associated with early-onset autoinflammatory disorders due to their impact on NF-kB signaling [23, 24].

Otulipenia, caused by homozygous loss-of-function mutations in the FAM105B gene, leads to continuous activation of NF-kB, resulting in early-life symptoms. Although extremely rare, otulipenia typically presents in the neonatal period with recurrent fevers, joint swelling, painful nodular red rashes, gastrointestinal inflammation with diarrhea, and failure to thrive. Skin biopsies in affected patients often reveal neutrophilic dermatosis and small to medium-vessel vasculitis. Laboratory findings usually show normal immune cell counts, immunoglobulin levels, and vaccine titers. Anti-TNF-α therapy has been successful in the few reported cases, while other therapeutic options have demonstrated limited efficacy [25].

A20 haploinsufficiency, an autosomal dominant disorder caused by mutations in the TNFAIP3 gene, also leads to systemic inflammation. Patients commonly experience symptoms such as oral and genital ulcers, uveitis, and fevers resembling familial Behcet’s disease. Other NF-kB-related disorders with similar inflammatory presentations may emerge later in life [26].

### Neonatal Kawasaki disease

Kawasaki disease (KD) is an acute febrile condition that can also affect neonates, with a higher incidence of incomplete Kawasaki disease (IKD) compared to older children. Neonatal KD may present with symptoms such as rash, changes in the extremities, and cervical lymphadenitis, even in the absence of fever. In a review of neonatal KD cases, IKD accounted for 68.4%, and coronary artery lesions (CALs) were observed in 89.5% of cases. Laboratory findings commonly include elevated CRP and platelet count. The etiology of KD remains unclear, but it may be associated with infections, including sepsis and pneumonia, especially in neonates. Early use of intravenous immunoglobulin (IVIG) appears beneficial, with the timely administration reducing the risk of CALs. Despite the absence of fever in some neonates, prompt recognition and treatment are crucial for improving outcomes. Neonates with KD require close monitoring, particularly for cardiovascular complications, to ensure a favorable prognosis [27].

### Neonatal Behçet’s disease

Behçet’s disease (BD) in the neonatal period is scarce, with only a limited number of case reports available. Postnatal manifestations may include orogenital ulcerations and pustulonecrotic skin lesions. Additionally, symptoms such as pyrexia and bloody diarrhea have been observed. In one study, aortic lesions were noted in a 16-week-old fetus from a mother with BD. The impact of BD on pregnancy outcomes remains poorly understood. A retrospective analysis of 61 pregnant women with BD, 23 of whom had active disease, concluded that BD does not pose a significant risk during pregnancy [20]. These cases have been observed in newborns of mothers with active BD, suggesting the possibility of perinatal transmission. Most of these cases resolved spontaneously without long-term complications [28, 29].

### Neonatal-onset juvenile ıdiopathic arthritis

Juvenile idiopathic arthritis is extremely rare in the neonatal period, as it typically manifests during childhood, following the newborn stage. However, there have been reported cases of very early-onset JIA. The condition can present with joint inflammation and systemic symptoms. In the diagnosis, it is crucial to rule out malignancies and infectious causes such as tuberculosis and brucellosis. DMARDs may be effective in treatment [30, 31].

## Conditions associated with maternal autoimmune rheumatologic diseases in neonates

### Maternal SLE and neonatal lupus

Research on mothers with SLE has revealed that the rate of preterm births is higher in these pregnancies, and the newborns are more likely to require neonatal intensive care. Babies born to mothers with active SLE have a higher incidence of prematurity, fetal loss, and asphyxia compared to those born to mothers with inactive SLE. It is believed that maternal proteinuria, hypertension, and renal involvement contribute to placental insufficiency, leading to these complications [6]. While maternal SLE contributes to complications related to placental insufficiency, the antibodies transferred from the mother, such as anti-Ro, anti-La, and anti-U1RNP, have significant consequences in neonatal lupus. These antibodies are present in nearly 40% of cases and play a role in the pathogenesis of neonatal lupus. Neonatal lupus affects around 1–2% of infants born to mothers who test positive for these antibodies, as it is transmitted to the fetus through the placenta [32]. Neonatal lupus can present with benign findings such as skin lesions, abnormal liver function tests, and cytopenia. However, it can also manifest with life-threatening conditions like cardiac arrhythmias, aplastic anemia, central nervous system (CNS) anomalies, and cholestasis. Table 1 shows the determination of involvements and approaches in neonatal lupus across the prenatal, antenatal, and postnatal periods.

**Table 1:** Involvements and approaches in neonatal lupus across prenatal, antenatal, and postnatal periods

Involvements	Approaches
Cutaneous	Usually, no treatment requiring Ultraviolet protection Applying sunscreen Low-potency topical corticosteroids when the risk of scarring or skin atrophy
Hematological	Usually asymptomatic, transient, and no treatment requiring In severe cases, - Blood transfusion - Corticosteroids at a dose of 1–2 mg/kg for 5 days or IVIG at a dose of 1 g/kg
Hepatobiliary	Transient and no treatment requiring In persistent cases, - Corticosteroids at a dose of 1–2 mg/kg for 5 days, then gradually decreased
Neurological	Transient and no treatment requiring In severe cases, - IVIG at the dose of 2 g/kg
Cardiac	Prenatal screening for anti-Ro/SSA and anti-La/SSB antibodies Perform fetal echocardiography every 1 to 2 weeks between the 18th and 26th weeks of pregnancy In the detection of heart block or endocardial fibroelastosis in the fetus, - Administer dexamethasone and IVIG to the mother Pacemaker application in neonates with complete AV block

*AV* atrioventricular, *IVIG* intravenous immunoglobulin

Benign cutaneous lesions typically appear within the first 6 weeks of life, though some may be present at birth. These lesions usually manifest as photosensitive erythematous annular lesions, often located on the scalp and around the eyes, and generally resolve without scarring by 15–17 weeks [32].

The most severe manifestations of neonatal lupus are related to cardiac involvement. Approximately 80–95% of all congenital heart block cases are associated with neonatal lupus. This is particularly common in mothers who are positive for anti-Ro/La antibodies. Anti-Ro antibodies bind to cross-reactive epitopes on calcium-regulating molecules, such as ion channels, leading to disturbances in calcium homeostasis and signal electrophysiogenesis at the atrioventricular (AV) node. Depending on the sensitivity of the fetal HLA genes, some cases may resolve, while others may develop permanent AV block due to fibrosis and calcification. In neonatal lupus, cardiac clinical features often include fetal bradycardia, congestive heart failure, premature beats, pericardial effusion, and tricuspid regurgitation. Although the cardiac anatomy is typically normal in maternal antibody–mediated neonatal lupus, valvular lesions are frequently observed. In later stages, dilated cardiomyopathy and endocardial fibroelastosis may develop. Most infants with congenital heart block require a pacemaker [33, 34].

Hematological abnormalities, such as anemia, neutropenia, thrombocytopenia, and rarely aplastic anemia, occur in 15% to 48% of neonatal lupus erythematosus cases [35, 36]. Anti-SSA/Ro and anti-SSB/La autoantibodies are associated with hematologic involvement, with anti-RNP antibodies potentially contributing to thrombocytopenia. While cutaneous manifestations typically appear around the second week postpartum (ranging from 5 to 30 days), systemic involvement may not be evident until 2 to 3 months. Close monitoring of hepatic and hematologic systems is crucial during this period, with treatment initiated as needed. Autoantibodies tend to resolve by a median of 10 months after birth [37].

Although less commonly observed compared to cutaneous and cardiac manifestations, elevations in liver function tests, mild hepatosplenomegaly, cholestasis, and hepatitis may also occur. CNS lesions, particularly calcifications in the basal ganglia, have been noted infrequently [38]. Macrocephaly and hydrocephalus have been reported rarely [39].

There is no specific method for in utero diagnosis. The diagnosis is typically established when the infant of an anti-Ro/La-positive mother develops postnatal heart block or skin lesions. Therefore, mothers with SLE who are anti-Ro/La positive should undergo fetal echocardiography and magnetocardiography between 16 and 26 weeks of gestation. However, a definitive diagnosis cannot be made with these methods, as the course of fetal heart block remains uncertain. Additionally, it has been shown that prolongations in AV conduction do not necessarily lead to higher degrees of block. Some biomarkers were studied; levels of cord C-reactive protein, NT-proBNP, MMP-2, uPA, uPAR, and plasminogen were higher in fetuses affected by cardiac neonatal lupus compared to those unaffected [40].

Fluorinated steroids (FS) may be considered for the treatment of prenatal neonatal lupus, though their efficacy remains debatable. As placental hormones neutralize most steroids during placental transfer, the treatment primarily aims to prevent reversible inflammation and avoid complete scarring. However, the effectiveness of FS in treating heart block is uncertain. High-dose FS is generally not recommended due to the lack of clear benefits and the potential for adverse effects in both the mother and fetus, including IUGR, oligohydramnios, maternal infections, osteoporosis, and diabetes [35].

### Maternal APL positivity/neonatal anti-phospholipid antibody syndrome

Antiphospholipid antibodies target the placenta by binding to β2-glycoprotein-I expressed on the surface of trophoblast cells. Up to 30–70% of patients with both SLE and aPL will develop APS after 20 years of follow-up. The incidence of aPL positivity among mothers with SLE varies. Anti-cardiolipin antibodies are positive in 12–44% of cases, lupus anticoagulants in 15–34%, and anti-β2-glycoprotein I (anti-β2-GPI) in 10–19% of cases. SLE-related APS is associated with higher rates of fetal loss and complications compared to primary APS. The presence of aPL is linked to poor pregnancy outcomes. Mothers testing positive for aPL antibodies frequently encounter early pregnancy complications. After the 10th week, risks include fetal death, premature birth, and placental insufficiency. Even with low disease activity, fetal involvement remains significant, increasing the risk of spontaneous abortion and premature birth [41].

In neonatal APS, various immune mechanisms involving neutrophils, monocytes, and platelets contribute to the disease’s development. Thrombocyte dysfunction and activation are critical in vascular inflammation associated with APS. Anti-β2-GPI antibodies can activate platelets by binding to apolipoprotein-E-receptor-2 and β2-glycoprotein-I-bα, leading to platelet dysfunction. This activation contributes to thrombus formation, a key feature of APS. Additionally, leukocyte-platelet complexes exacerbate vascular inflammation. The interaction of platelets with other immune cells, such as monocytes, which express pro-inflammatory cytokines and tissue factors, further amplifies the risk of thrombosis. Genetic factors, including mutations associated with thrombophilia, also contribute to the increased risk of thrombotic events in APS patients.[39–42]. The clinical spectrum of neonatal APS is broad, ranging from asymptomatic aPL positivity to severe thrombotic events. The most commonly observed clinical features include thrombosis, intracranial hemorrhage, IUGR, prematurity, and SGA. Hematological abnormalities may manifest as thrombocytopenia, lymphopenia, and autoimmune hemolytic anemia. Additional findings may include livedo reticularis, Raynaud’s phenomenon, and skin ulcers. Cutaneous manifestations may represent the initial and sole presentation of APS. Catastrophic APS is a rare and life-threatening variant in neonates, characterized by multi-organ failure and microvascular thromboses. Unlike in adults, cardiac lesions are rarely observed in neonates [41].

In the treatment of neonates, the goal is to correct arterial and venous thromboses and to prevent their recurrence. Low-dose aspirin is the first-line treatment. Low-molecular-weight heparin (LMWH) and vitamin K antagonists can be administered for long-term anticoagulation therapy. Despite long-term treatment, recurrent thromboses are common. INR measurement for warfarin dose adjustment is influenced by aPL positivity, leading to falsely elevated INR values. Recent studies on antimalarial therapy have shown promising results. In SLE-associated APS, managing the underlying disease is crucial. In cases of catastrophic APS, treatment may include intravenous immunoglobulin, plasmapheresis, steroids, and rituximab [41–44].

### Maternal primary Sjögren syndrome

Primary Sjögren syndrome is usually seen in women in their fourth and fifth decades, and although rare, cases of pSS during pregnancy have been reported in the literature. Preterm birth and SGA are particularly associated with this condition. Congenital heart block is a significant cause of morbidity and mortality in infants of mothers with pSS, as anti-Ro/La antibodies begin to cross the placenta at approximately the 12th week of gestation, affecting the AV node. Maternal autoantibodies can activate fibroblasts in the fetal heart, leading to fibrosis through the TGF-β signaling pathway. Specific HLA class II genotypes, like HLA-DR3 and HLA-DR2, increase the risk of developing pSS and may enhance the effects of these maternal autoantibodies on the fetus.

Additionally, abnormal microRNA expression in mothers with pSS, particularly miR-146a and miR-155, can worsen inflammation and autoimmune responses. These changes can impact fetal development, leading to complications such as neonatal lupus and congenital heart block [45]. Postnatal pacemaker implantation is recommended in infants with heart block [46]. Treatments such as steroids, intravenous immunoglobulin (IVIG), plasmapheresis, and beta sympathomimetics such as terbutaline can be tried during pregnancy. However, the effectiveness of these treatments has not been definitively established [47].

### Maternal rheumatoid arthritis

In mothers with rheumatoid arthritis, preterm birth and SGA are more common compared to the general population. The adverse effects of drugs used in RA on the fetus are more important than the disease itself. The impact of maternal antirheumatic drugs on the newborn is detailed in the further section [48].

### Maternal dermatomyositis/polymyositis

Studies demonstrating the effects of maternal dermatomyosites\polymyosities on neonates are limited. What is known is that postnatal outcomes are favorable in those with inactive disease during pregnancy and fetal loss, SGA, and preterm birth in those with active disease. Maternal anti-Jo-1, anti-SRP, and anti-MDA5 antibodies can cross the placenta and damage fetal muscle and skin cells. These maternal autoantibodies can disrupt MyoD, myogenin, and other myogenic regulatory factors that play a role in fetal development, impairing fetal muscle development and muscle weakness. Disruption of the myogenic program in fetal muscle cells can lead to congenital myopathy and muscle weakness in the postnatal period. While steroids and IVIG can be used, the benefits of tumor necrosis factor (TNF) inhibitors have not been demonstrated [45–47].

### Maternal systemic sclerosis

In maternal systemic sclerosis cases, there is an increased incidence of preterm birth and SGA infants. Patients with widespread cutaneous SSc and those positive for anti-Scl-70 antibodies and/or anti-RNA polymerase III antibodies are particularly at heightened risk. A characteristic feature of SSc is pronounced endothelial dysfunction in small blood vessels and excessive fibrosis development. In maternal SSc, autoantibodies can bind to fetal endothelial cells, leading to apoptosis and dysfunction in these cells. This process results in vascular lumen narrowing and impaired blood flow [49]. Increased expression of TGF-β in maternal SSc can also trigger fibrotic responses in fetal tissues, adversely affecting fetal lung development and potentially leading to complications such as pulmonary hypertension in the postnatal period [50]. Maternal scleroderma renal crisis is a life-threatening condition that can cause fetal renal dysfunction. Treatment may include steroids, azathioprine, hydroxychloroquine, calcium channel blockers, and LMWH [48].

## Conditions associated with the use of anti-rheumatic medications during pregnancy

Effective management of rheumatic diseases during pregnancy is crucial to prevent joint or organ damage and minimize the negative impact of the disease on pregnancy outcomes. Each patient is required to undergo a thorough individual assessment to control disease activity while reducing the use of medications that may be harmful to the mother or fetus. It is essential to plan for pregnancy when the disease has been inactive for at least 6 months and the mother is taking non-teratogenic medications [51]. In this section, we aim to explain the pregnancy categories of commonly used rheumatologic drugs and their potential effects on the fetus and newborn (Table 2).

**Table 2:** Pregnancy categories and potential effects of commonly used rheumatologic drugs on the fetus and newborn

Drugs	Pregnancy categories	Potential effects	Additional explanations
Non-steroidal anti-inflammatory drugs	B	Premature closure of ductus arteriosus Oligohydramnios Placental insufficiency	Use should be avoided in the third trimester given the risks
Disease-modifying antirheumatic drugs			
Methotrexate	X	Severe teratogen	Must be stopped 3 months before conception. Folic acid supplementation suggested
Leflunomide	X	Severe teratogen	It can take up to 2 years to fully eliminate this medication from the body. cholestyramine wash-out is recommended
Azathioprine	D	Prematurity Encephalocele, sternocleidomastoid anomalies Congenital cataracts Atrial and ventricular septal defects	
Colchicine	C	Prematurity, small gestational age	
Hydroxychloroquine	C	Small gestational age	
Sulphasalazine	B	Transient neutropenia Cleft palate Macroglossia, congenital deafness	Folic acid supplementation recommended
Mycophenolate mofetil	D	Miscarriage	
Biologic DMARDs			
*Anti-tumor necrosis factors*	B	No increased rate of miscarriage or congenital malformations when used during the 1st trimester	After 3rd trimester, close follow-up
*Canakinumab*	C	Fetal skeletal, development delayed in animal models	
*Anakinra*	B		No harm was demonstrated in animal studies
*Tocilizumab*	C	No teratogenicity in animal models at any dose	Discontinued 10 weeks before a planned pregnancy
*Rituximab*	C	Miscarriage Congenital and fetal hematologic abnormalities	
*Corticosteroids*	D	Congenital malformations (cleft lip palate) Miscarriages	Use the lowest effective dosage during pregnancy
*Intravenous immunoglobulin*	C	No reports of fetal malformations in humans	

Nonsteroid anti-inflammatory drugs (NSAIDs) can be used cautiously during pregnancy, especially before the third trimester when the organogenesis is still developing. After the third trimester, they may pose risks such as premature ductus arteriosus closure, placental problems, and oligohydramnios. NSAIDs are generally categorized as B in terms of pregnancy safety. Corticosteroids are safe to use during pregnancy, but prolonged use of high doses (over 15 mg/day) can increase the risk of early membrane rupture and preterm labor. High doses may also elevate the risk of oral clefts and palate defects. It is important to use the lowest effective dose to minimize these risks.

Disease-modifying anti-rheumatic drugs like methotrexate (MTX) and leflunomide are known to cause birth defects and are considered unsafe during pregnancy. Azathioprine is categorized as D. Colchicine and hydroxychloroquine fall under category C, while sulfasalazine is categorized as B. Despite the absence of randomized controlled trials, several observational and retrospective studies in patients with familial Mediterranean fever have found that colchicine use during pregnancy is beneficial for disease management without increasing the risk of miscarriage or congenital malformations. Although higher rates of preterm delivery and lower median birth weight have been associated with colchicine use, underlying rheumatic disease may also contribute to these adverse outcomes. Hydroxychloroquine is not related to congenital abnormalities or malformations and can be continued throughout pregnancy. In pregnancies of anti-Ro/La positive mothers, their immunomodulatory and anti-platelet effects may be beneficial in managing neonatal lupus. Hydroxychloroquine can be particularly effective in patients with antiphospholipid syndrome (APS) who are aPL-positive [34]. Sulphasalazine has been used for many years to manage arthropathies and inflammatory bowel disease during pregnancy [52].

Biological disease–modifying antirheumatic drugs (DMARDs) are generally safe during pregnancy. Drugs like adalimumab, anakinra, etanercept, infliximab, secukinumab, and ustekinumab are categorized as B. Others, including abatacept, apremilast, belimumab, canakinumab, rituximab, tocilizumab, and tofacitinib, fall under category C, indicating a higher potential risk. Common side effects of these medications during pregnancy include SGA babies and preterm birth. Some animal studies have linked canakinumab to skeletal issues, while rituximab has been associated with congenital heart defects and blood-related issues. In a report of 40 patients taking another anti-IL-1 drug, anakinra, during pregnancy, no increased risk of miscarriage or congenital malformations was observed [53]. TNF inhibitors can remain in the newborn’s system for up to 6 months after birth, so live vaccines should be avoided [52].

Intravenous immune globulin is compatible with pregnancy and is utilized for various indications, including primary or secondary antibody immunodeficiencies, dermatomyositis, and APS. IVIG is also compatible with breastfeeding. In humans, IVIG appears to cross the placenta significantly after 30 to 32 weeks of gestation, even when modifications alter the Fc-binding sites. To date, there have been no reports of fetal malformations associated with IVIG use [54]. In patients with SLE who experience recurrent spontaneous abortions and are treated exclusively with IVIG during pregnancy, maternal and fetal outcomes have been demonstrated to be both successful and safe. Clinical and laboratory data, as well as successful delivery, have been observed in these cases [55].

Antiviral antibodies in IVIG can hinder the effectiveness of live attenuated vaccines by reducing their ability to replicate. Therefore, waiting 8–11 months after high-dose IVIG is advised before getting a live attenuated virus vaccination. However, earlier vaccination may be preferred in cases of measles outbreaks to provide some immunity [56]. Neonates exposed to biologic DMARDs do not need to change their vaccination schedule. The previous recommendation of waiting 6 months before vaccination has been updated, and now, the only recommendation is to delay the tuberculosis vaccine [57]. Infants born to mothers receiving TNF inhibitors may receive the rotavirus vaccine with caution. However, if the mothers have had rituximab, the vaccination may be delayed [56].

## Conclusions

The clinical presentation of rheumatic diseases in newborns is highly diverse, with symptoms potentially affecting various organ systems, including the skin, joints, heart, and lungs. In some cases, newborns may exhibit non-specific symptoms such as fever, rash, and poor feeding, underscoring the importance of early diagnosis and appropriate management. In instances with prolonged fever, rash, and joint involvement with no identifiable cause, it is advisable to seek consultation from a pediatric rheumatologist to assess the possibility of autoinflammatory diseases (Box 2).

**Box 2** Best practice for autoinflammatory diseases with perinatal onset

**Table:** 

Autoinflammatory diseases with perinatal onset	
When should autoinflammatory disease in a neonate be suspected?	What to do if autoinflammatory disease is suspected in a neonate?
• In a neonate with unexplained fevers, • Presence of systemic inflammation without evidence of infection • Irritability, poor feeding, and skin rash and lesions	• Exclusion of possible underlying infectious, metabolic, endocrinological and hematological causes • Consideration genetic testing for autoinflammatory disorders • Early initiation of anti-inflammatory therapies to suppress systemic inflammation to prevent morbidity and mortality • Referral to a specialist for assessment of positive genetic testing results and treatments

Disease severity, the transfer of maternal autoantibodies through the placenta, and the potential effects of maternal therapy are risk factors for the fetus and neonate. To achieve the best possible outcomes for both mother and infant, a careful, multidisciplinary approach involving an obstetrician, a rheumatologist, and a neonatologist is essential.

## Data Availability

No datasets were generated or analysed during the current study.

## Abbreviations

Anti-β2-GPI: Anti-β2-glycoprotein I
aPL: Antiphospholipid antibodies
APS: Antiphospholipid syndrome
AV: Atrioventricular
CAPS: Cryopyrin-associated periodic syndromes
CINCA: Chronic infantile neurological cutaneous and articular syndrome
DITRA: Interleukin-36 receptor antagonist deficiency
DM/PM: Dermatomyositis/polymyositis
FCAS: Familial cold autoinflammatory syndrome
FS: Fluorinated steroids
IL: Interleukin-1
IUGR: Intrauterine growth restriction
IVIG: Intravenous immunoglobulin
JAK: Janus kinase
JIA: Juvenile idiopathic arthritis
LMWH: Low-molecular-weight heparin
MWS: Muckle-Wells syndrome
NOMID: Neonatal-onset multisystem inflammatory disease
pSS: Primary Sjögren’s syndrome
RA: Rheumatoid arthritis
SGA: Small for gestational age
SLE: Systemic lupus erythematosus
SSc: Systemic sclerosis
TNF: Tumor necrosis factor
